# A Case of Postoperative Biliary Leak in a Patient With Duplicated Cystic Ducts

**DOI:** 10.7759/cureus.55854

**Published:** 2024-03-09

**Authors:** Giri Movva, Jordan C Malone, Jaison S John, Patrick D Sweet

**Affiliations:** 1 Department of Internal Medicine, University of Texas Medical Branch, Galveston, USA; 2 Department of Gastroenterology, Hunt Regional Medical Center, Greenville, USA

**Keywords:** bile system, duplicated bile ducts, bile leak, biloma, bile duct injury

## Abstract

Duplicated cystic ducts are a rare congenital malformation with less than 20 reported cases before 2019. This malformation is important to identify to reduce the risk of intraoperative complications such as bile duct injuries that can increase postoperative morbidity and mortality. We present the case of a 62-year-old male with duplicated cystic ducts that were ligated during laparoscopic cholecystectomy and subsequently complicated by postoperative biloma formation. Treatment options for biliary leak include endoscopic retrograde cholangiopancreatography (ERCP) with stenting, percutaneous drainage, and duct embolization. Each carries the risk of complications such as infection, duct perforation, and stent/drain displacement. Roux-en-Y hepaticojejunostomy (RHYJ) tends to be the last resort when other minimally invasive procedures fail. It is imperative to identify postoperative complications related to cystic duct anomalies and the various treatment options available should these complications occur.

## Introduction

Duplicated cystic ducts with a single gallbladder, with less than 20 reported cases before 2019, are a rare congenital malformation that is typically diagnosed intraoperatively or postoperatively due to complications [1-4]. It is important to identify these anomalies in the periprocedural setting to reduce the risk of postoperative complications, including bile duct injuries and subsequent biliary leaks that increase postoperative morbidity and mortality [1-6]. Biliary leaks can lead to the formation of bilomas. Bilomas are infrequently encountered and described as an encapsulated collection of bile inside or outside the biliary system but within the abdominal cavity [5]. Bilomas are commonly seen with trauma, surgery, or minimally invasive procedures within the biliary system [5]. Cystic duct injuries contribute to more than 50% of bilomas [5]. Here, we present the case of an older male with duplicated cystic ducts who underwent cholecystectomy with a subsequent postoperative biliary leak and biloma formation. We discuss the imaging utilized to make this diagnosis as well as discuss the challenges in diagnosis and treatment associated with bile duct injury in a patient with a rare congenital abnormality.

## Case presentation

A 62-year-old male with a past medical history significant for biliary dyskinesia status-post cholecystectomy was transferred from an outside hospital (OSH) to our institution with symptoms of non-radiating epigastric pain, nausea, vomiting, and chills.

**Figure 1:**
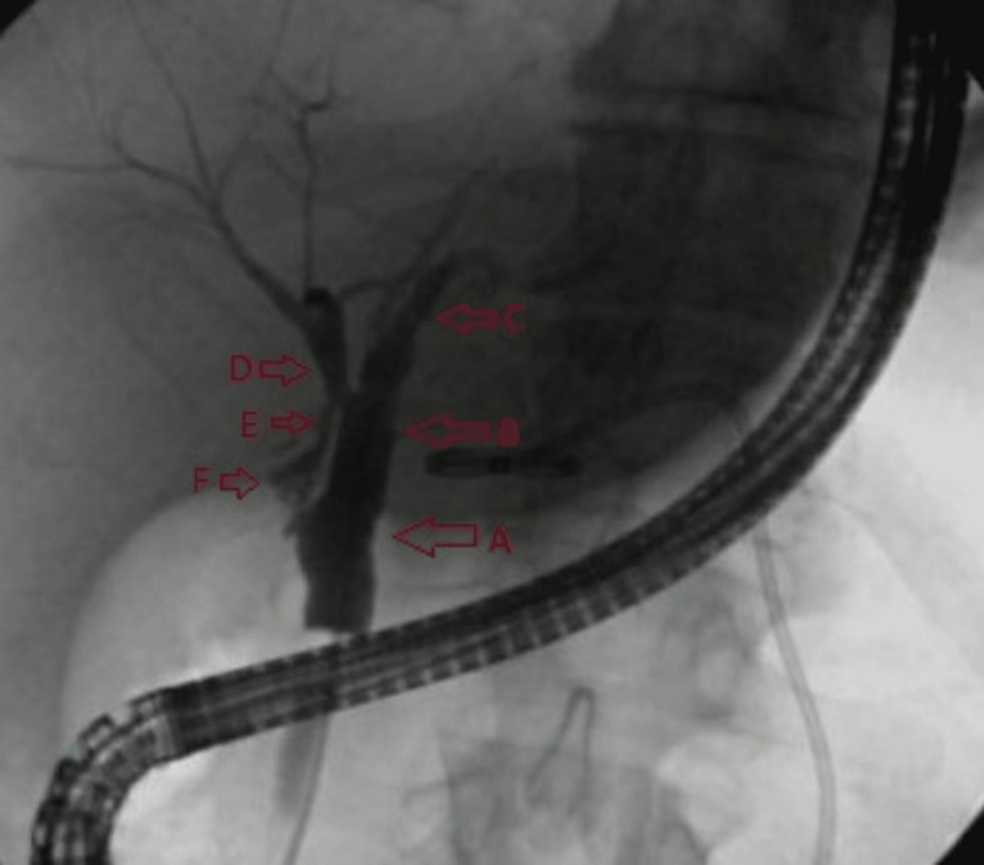
Cholangiogram A: Common bile duct. B: Common hepatic duct. C: Left hepatic duct. D: Right hepatic duct. E and F: Cystic ducts

Pertinent interval history includes an OSH laparoscopic cholecystectomy two months prior to his second presentation at our home institution. Intraoperatively, the patient was found to have duplicated cystic ducts that were ligated. Subsequent computed tomography (CT) of the abdomen showed a bile leak with biloma formation posterior to the left lobe of the liver extending into the gallbladder fossa for which he underwent endoscopic retrograde cholangiopancreatography (ERCP). ERCP revealed papillary stenosis with common bile duct dilation but no apparent leak on the cholangiogram. A plastic biliary stent was placed, and the fluid collection was drained. One month after OSH cholecystectomy, he presented with a recurrence of abdominal pain at our home institution. Magnetic resonance imaging (MRI) showed a persistent fluid collection near the gallbladder fossa. A hepatobiliary iminodiacetic acid scan was performed and showed a persistent biliary leak into the subhepatic space. The patient underwent repeat ERCP with a normal cholangiogram (Figure 1), and the plastic biliary stent was replaced with a metallic stent.

A right-sided percutaneous transhepatic cholangiogram was performed to investigate a possible right hepatic ductal leak. This showed contrast extravasation into an extra-hepatic collection adjacent to the preexisting drain. An external drain was placed into the fluid collection, and the patient's symptoms subsequently improved.

**Figure 2:**
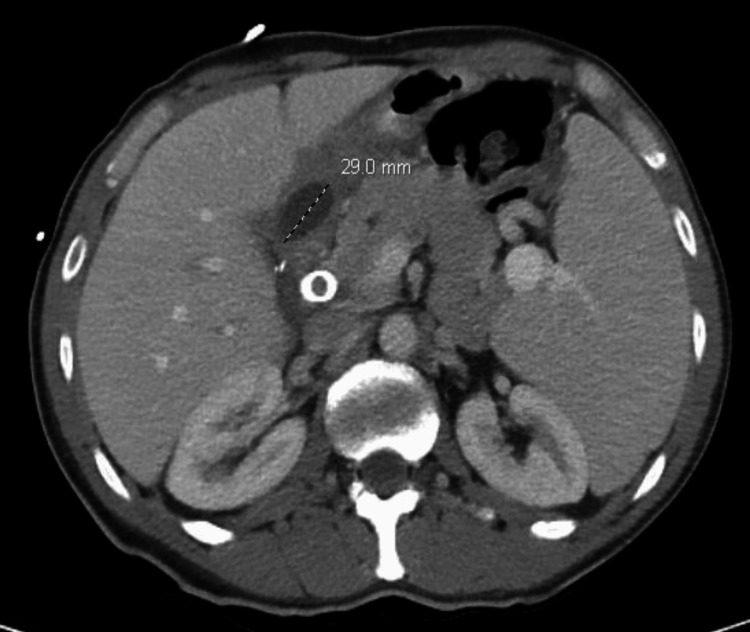
CT of the abdomen and pelvis with contrast (axial view): a 2.9 cm fluid collection near the right subhepatic region CT: computed tomography

On his second presentation at our institution, lab work was significant for alkaline phosphatase of 344 (34-122 U/L) and lipase of 606 (0-220 U/L). CT showed the development of a 2.9 cm fluid collection (Figure 2) adjacent to the subhepatic drain tip.

The patient underwent biliary drainage interrogation that demonstrated right-sided biliary ducts with no communication with the common bile duct (CBD); subsequent attempts to recanalize the transhepatic drainage tract were unsuccessful. He underwent magnetic resonance cholangiopancreatography (MRCP), which revealed a perihepatic fluid collection that measured 2x3.8x2.6 cm with communication to the right-sided bile duct. A cholangiogram of the right posterior external biliary drain demonstrated no filling of the CBD from the right-sided access. However, frank communication with the gallbladder fossa collection was compatible with a bile leak. Attempts for catheterization of the bile leak site via a right biliary ductal approach were made but unsuccessful, with subsequent dissection of ducts into the hepatic parenchyma limiting further intervention. A new external biliary drain was placed. Future attempts were postponed, allowing for the healing of the dissection. The patient subsequently developed a fever for which intravenous piperacillin-tazobactam was started. Blood cultures grew pan-susceptible *Escherichia coli*, and he completed a course of targeted intravenous antibiotics. A multidisciplinary meeting to discuss approaches for bile leak intervention led to a tentative plan for right-sided bile duct embolization with coils. The patient underwent right-sided bile duct embolization, but attempts to cross the right-sided bile duct into the suspected leak were unsuccessful due to persistent duct dissection. The patient was discharged to undergo outpatient right-sided bile duct embolization in three to four weeks to allow for bile duct healing at a different institution due to changes in his insurance.

## Discussion

While duplicated cystic ducts are rare, bile duct injury in patients who undergo laparoscopic cholecystectomy is a common complication that can lead to postoperative complications reported at rates of 0.2-2%, which we postulate is likely higher in patients with congenital biliary anomalies compared to the general population [1,3]. Many diagnostic modalities have been implemented preoperatively to help diagnose cystic duct anomalies, namely, ERCP, endoscopic ultrasound, CT, and MRCP [1]. Unfortunately, cystic duct anomalies are oftentimes not properly identified on preoperative imaging [2-4]. According to Anisi et al., multiple studies have attempted to determine ideal diagnostic imaging for cystic duct anomalies [1]. One such study was Munie et al., which summarized 20 case reports of duplicated cystic ducts from 1961 to 2019. Of the seven patients who underwent preoperative imaging, only three were diagnosed with malformations during preoperative ERCP [1,2]. The majority of patients were diagnosed intraoperatively [2]. This supports the idea that preoperative imaging is needed, though even if negative, it is essential to be aware of these variations during surgery. Sarawagi et al. studied imaging features of cystic duct variations in 198 patients using MRCP. They concluded that MRCP was an ideal imaging technique for determining cystic duct anatomy preoperatively, as they could assess cystic duct insertion to the CBD with this modality. No duplicate cystic ducts were discovered in the 198 patients; thus, they are not completely applicable to our case [1,7].

The ideal treatment for bile duct injuries is when they are discovered intraoperatively. It is important to monitor patients postoperatively for bile leak, even if biliary injury was found and treated intraoperatively [4]. A common treatment for postoperative bile leak is ERCP with stent placement, which helps facilitate bile flow, allowing passive leak healing [5,6,8]. If an endoscopic intervention fails, percutaneous intervention, such as external biliary drain placement, can be performed [5,8-10]. Alternative options, such as cystic duct embolization with vascular coils and micropledgets, have been proposed when direct access to the biliary leak is available [5,8,10]. Complications of these minimally invasive procedures include but are not limited to infection, duct perforation, hemorrhage, or stent/drain displacement. However, if all are unsuccessful, the Roux-en-Y hepaticojejunostomy (RYHJ) procedure is the definitive surgical treatment for biliary ductal injuries [9,11]. Complications of RYHJ can include but are not limited to anastomotic strictures, anastomotic leak, cholangitis, and the risk of requiring further surgical intervention [11]. With time, the RYHJ procedure has evolved, thus decreasing the risk of postoperative complications, especially with the transition to a laparoscopic approach [11].

In our patient, the duplicated cystic ducts were visualized during his cholecystectomy and ligated intraoperatively. Though the literature has mentioned that multiple diagnostic modalities can be utilized prior to surgery to evaluate the biliary anatomy, cystic duct variations are not always seen. Our patient had a postoperative complication with biloma formation. With multiple CT scans, ERCPs, and cholangiograms, we were not able to determine the communication of the biloma, though the biloma was visible. However, with MRCP, we could see that the right-sided bile duct communicated with the biloma. The patient underwent multiple percutaneous drainages with ERCP for stent placement. Due to his persistent bile leak, embolization of the right hepatic duct was attempted, which was complicated by duct dissection. The duct dissection was the likely result of multiple manipulation attempts with prior ERCPs in short intervals.

## Conclusions

This case demonstrates the need for physician awareness regarding cystic duct anomalies in patients even if diagnostic imaging is negative. Close monitoring postoperatively is important for possible biliary ductal injury even if biliary injury is treated intraoperatively. It is imperative to be aware of treatment options such as minimally invasive procedures, thus minimizing the potential for larger-scale, invasive interventions such as the RYHJ. We want to bring awareness to the diagnostic and therapeutic challenges related to biliary injury in a patient with a congenital biliary abnormality. These cases can be costly to the patient as well as the healthcare system if not properly diagnosed and managed.
